# Rehabilitation exoskeleton torque control based on PSO-RBFNN optimization

**DOI:** 10.1371/journal.pone.0285453

**Published:** 2023-08-08

**Authors:** Jiayi Li, Yuanzheng Tai, Fanwei Meng

**Affiliations:** School of Control Engineering, Northeastern University at Qinhuangdao, Qinhuangdao, China; University of Bradford, UNITED KINGDOM

## Abstract

Exoskeletons are widely used in the field of medical rehabilitation, however imprecise exoskeleton control may lead to accidents during patient rehabilitation, so improving the control performance of exoskeletons has become crucial. Nevertheless, improving the control performance of exoskeletons is extremely difficult, the nonlinear nature of the exoskeleton model makes control particularly difficult, and external interference when the patient wears an exoskeleton can also affect the control effect. In order to solve the above problems, a method based on particle swarm optimization (PSO) and RBF neural network to optimize exoskeleton torque control is proposed to study the motion trajectory of nonlinear exoskeleton joints in this paper, and it is found that exoskeleton torque control optimized by PSO-RBFNN has faster control speed, better stability, more accurate control results and stronger anti-interference, and the optimized exoskeleton can effectively solve the problem of difficult control of nonlinear exoskeleton and the interference problem when the patient wears the exoskeleton.

## Introduction

Rehabilitation exoskeletons play an important role in the medical field [1], and they have been widely used in recent years for the purpose of assisting medical staff to provide rehabilitation treatment for patients. According to the American Heart Management Association [2], approximately 700,000 people worldwide suffer from stroke each year, and 90 percents of those who survive have some degree of impairment in motor function. Rehabilitation exoskeleton, which plays an important role in the rehabilitation of patients [3], can help sports injury patients to perform fixed gait training and re-exercise damaged nerves. The control performance of the exoskeleton plays a key role in the effectiveness of rehabilitation, GM Gasparri et al. [4] conducted in-depth research on exoskeleton ankle joint torque control to ensure that the exoskeleton can meet the changing biomechanical needs of the patient while walking; Yu et al. [5] studied exoskeleton PID control in order to reduce the uncertain steady-state error in exoskeleton control; X Li et al. [6] performed BP neural network optimization on exoskeleton, and verified the good performance of the optimized exoskeleton in joint torque estimation; Y Long et al. [7] used GA genetic algorithm to optimize exoskeleton control and make exoskeleton control more stable. Although the control performance of exoskeletons has been improved in the above research, the optimization of complex nonlinear exoskeleton control models is still difficult. In view of this nonlinear model that is difficult to optimize, this paper proposes an optimization strategy of PSO particle swarm algorithm combined with RBF neural network joint optimization, which combines the advantages of neural network for nonlinear system tuning and PSO algorithm acceleration tuning, so as to optimize the torque control of lower limb rehabilitation exoskeleton. Based on the above research, PSO particle swarm algorithm combined with RBF neural network is used to optimize the torque control of the lower limb rehabilitation exoskeleton. Q Zhang et al.’s research on non-Gaussian stochastic systems control theory and its application [8] and observer-based parameteric decoupling controller design [9] are also of guiding significance to this paper. Particle swarm algorithm (PSO) was first proposed by R Eberhart and J Kennedy [10], which is a population-assisted random search algorithm developed to study the foraging of birds, and continuously seeks the local optimal solution through the population particles to obtain the global optimal solution. In this paper, the PSO algorithm is used to optimize the weights of RBF neural networks and seek the optimal solution with the lowest error of exoskeleton joints. The RBF neural network [11] was first proposed by J Moody and C Darken as a neural network structure that mimics feelings in the human brain. Aiming at the nonlinear control of the exoskeleton system in this paper, the local optimization characteristics of the RBF neural network optimized by the PSO algorithm can enable it to find the optimal control parameters of the exoskeleton more quickly and accurately, thereby reducing the error and the influence of disturbance, so that the output trajectory of the exoskeleton joint can conform to the actual trajectory of the patient when walking. This paper is divided into three parts, the first part describes the research method, and analyzes the overall logic of optimizing the exoskeleton torque control of PSO-RBFNN. The second part is the qualitative analysis and quantitative analysis of the research results, the results show that the actual trajectory of the exoskeleton joint optimized by PSO-RBFNN can track the desired trajectory well, compared with exoskeleton PID control, the exoskeleton control performance based on PSO-RBFNN optimization torque control is superior. The third part presents the conclusions of the study.

## Materials and methods

### Overall structure of exoskeleton

**Fig 1:**
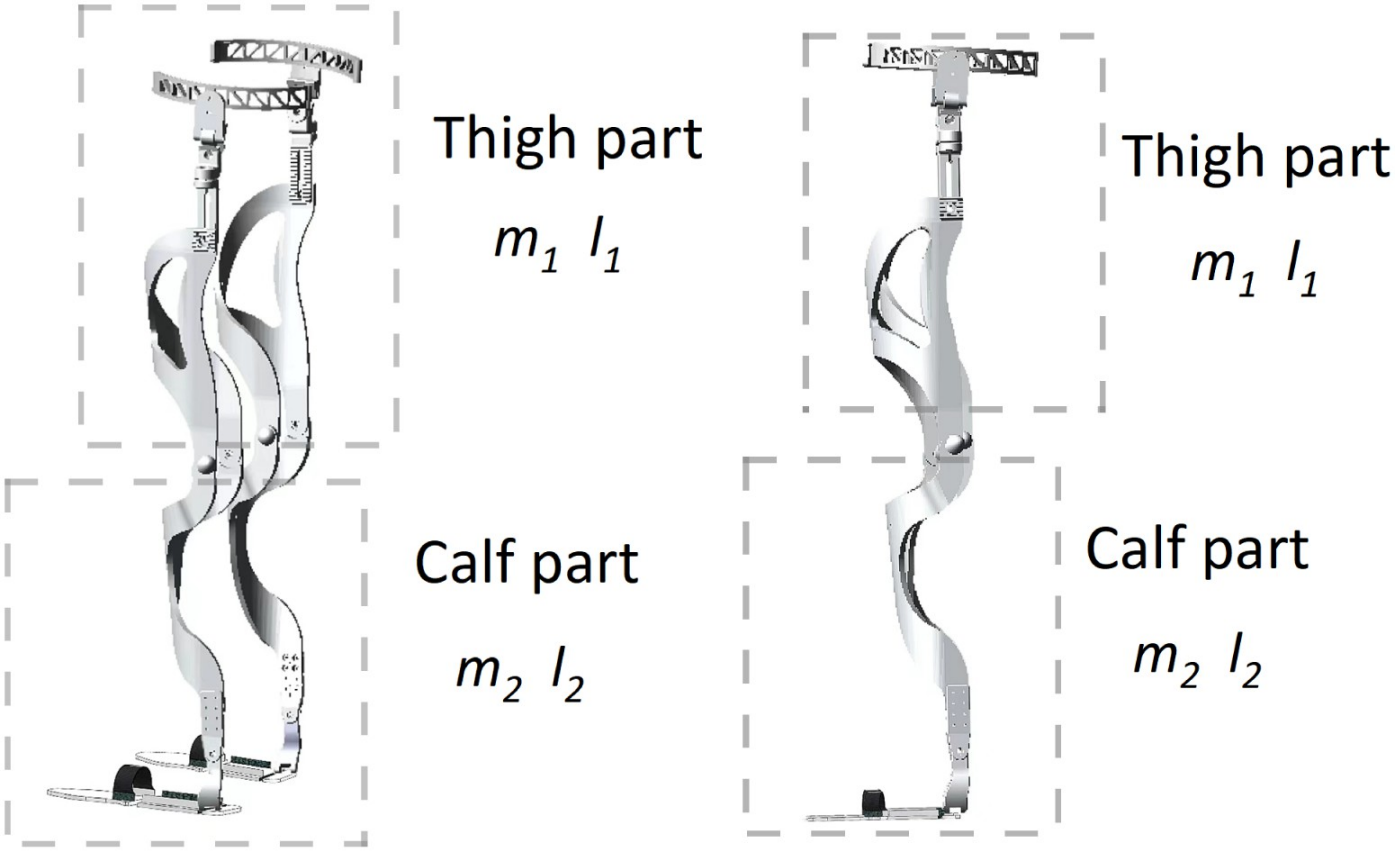
Exoskeleton shape structure.

Fig 1 shows the overall structure of the exoskeleton. The exoskeleton is composed of carbon-brazing dimensions and is lightweight overall. The exoskeleton is driven by a motor that is installed at both the hip and knee joints. The drive motor adopts the Maxson EC Flat series motor, which has the characteristics of high torque density and small size, and its maximum torque is 500 Nm, which is suitable for exoskeleton joint control [12]. The sensor is used to detect the joint angle and angular velocity in real time, and it is transmitted to the control system for real-time control. The specific parameters of the exoskeleton are shown in Table 1:

**Table 1:** Exoskeleton specific parameters.

Thigh mass (*m*_1_)	Calf mass (*m*_2_)	Thigh length (*l*_1_)	Calf length (*l*_2_)
5.25 kg	4.37 kg	0.5 m	0.4 m

Table notes the specific parameters of the exoskeleton.

As shown in Table 1, the overall weight of the exoskeleton is 21.04 kg, and Thigh Mass is the single leg weight from hip to knee, Calf Mass is the weight of single leg from knee to ankle, and the total length of the exoskeleton leg is centered on 0.5 m and 0.4 m, and can be retracted by 0.05 m, so as to meet the needs of different people.

In the coordinated movement of exoskeleton and human body, flexible materials are used to fix the exoskeleton and human legs, the battery and controller are placed at the waist and fixed by hard belt, and the drive motor is used to output different joint torques to achieve real-time control. The purpose of the exoskeleton is to provide lower limb rehabilitation training for people with walking disabilities, and a single exoskeleton lower limb has a total of 4 degrees of freedom [13], namely hip flexion and extension degrees of freedom (active), hip internal rotation degrees of freedom (passive), knee flexion and extension degrees of freedom (active), ankle flexion and extension degrees of freedom (passive). The drive motor is added to the active degree of freedom, and the input angle is adjusted in real time through the controller to ensure that the human body carries out walking rehabilitation training according to a fixed gait.

### Derivation of kinetic equations

The derivation of kinetic equations is the basis for real-time control of the system and deriving the dynamic equations of the exoskeleton system can optimize the performance indicators of the exoskeleton controller. In this paper, the torque control is optimized for the purpose of optimization, so it is particularly important to derive the kinematic equations.

**Fig 2:**
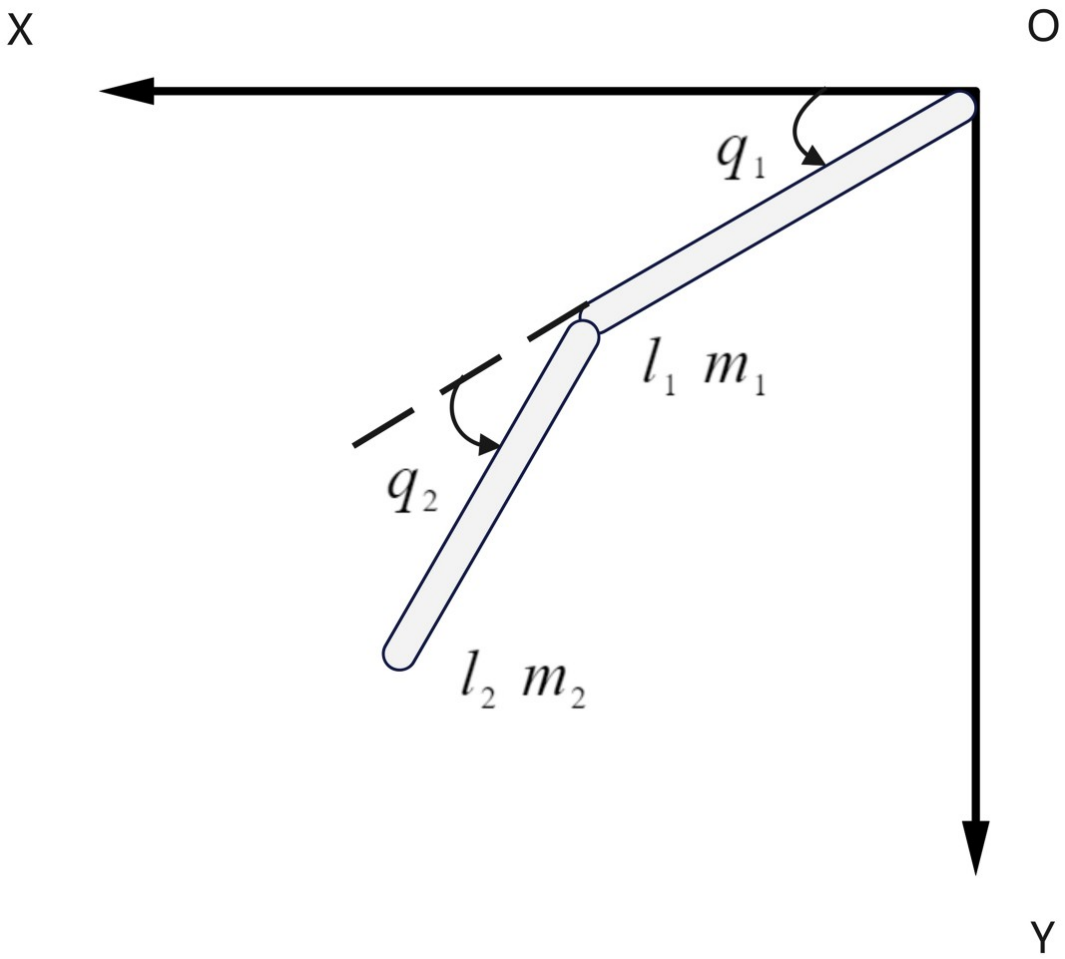
Single-leg simplified diagram.

Fig 2 is a simplified diagram of the single leg of the exoskeleton, and this paper uses the right lower limb of the exoskeleton as an example. The hip and knee joints are active joints, and the ankle joints are passive joints. The motor output torque at the active joint drives the leg movement of the exoskeleton, so the right lower limb can be simplified to the two-link form shown in Fig 2 [14], and the left lower limb is similar to the right lower limb.

As shown in Fig 2, *q*_1_, *l*_1_ and *m*_1_ are the thigh flexion and extension angle, length and mass, *q*_2_, *l*_2_ and *m*_2_ are the lower leg flexion and extension angle, length and mass. Assuming that the exoskeleton density is evenly distributed, and the center of mass of the thigh and calf is located in the center part of each linkage, the Lagrange kinetic equation [15] is derived from the exoskeleton to derive the relationship between the motor output torque and the angle of the leg joint.

### Derivation of kinetic energy

Suppose the coordinates of thigh center of mass and calf center of mass to (*x*_1_, *y*_1_), (*x*_2_, *y*_2_), the distance between the two centers of mass from the hip and knee joints is *d*_1_ and *d*_2_, and the sum of the squared coordinates of the two centers of mass coordinates can be obtained:

$$\begin{array}{c} \begin{array}{c} x_{1}^{2}+y_{1}^{2}={\left(d_{1}q_{1}\right)}^{2} \end{array} \end{array}$$ (1)

$$\begin{array}{c} \begin{array}{c} {x_{2}}^{2}+y_{2}^{2}={\left(l_{1}q_{1}\right)}^{2}+{\left[d_{2}{\left(q_{1}+q_{2}\right)}^{2}\right]}^{2}+2l_{1}d_{2}\left(q_{1}^{2}+q_{1}q_{2}\right)cosq_{2} \end{array} \end{array}$$ (2)

The kinetic energies *E*_*k*1_ and *E*_*k*2_ of the exoskeleton thigh and calf are:

$$\begin{array}{c} \begin{array}{c} E_{k1}=\frac{1}{2}m_{1}{\left(d_{1}q_{1}\right)}^{2} \end{array} \end{array}$$ (3)

$$\begin{array}{c} \begin{array}{c} E_{k2}=\frac{1}{2}m_{2}{\left(l_{1}q_{1}\right)}^{2}+\frac{1}{2}m_{2}{\left[d_{2}\left(q_{1}+q_{2}\right)\right]}^{2}+m_{2}l_{1}d_{2}\left(q_{1}^{2}+q_{1}q_{2}\right)cosq_{2} \end{array} \end{array}$$ (4)

The total kinetic energy of the exoskeleton single leg is:

$$\begin{array}{c} \begin{array}{c} E_{k}=E_{k1}+E_{k2} \end{array} \end{array}$$ (5)

### Derivation of potential energy

The potential energy *E*_*p*1_ and *E*_*p*2_ of the exoskeleton thigh and calf are:

$$\begin{array}{c} \begin{array}{c} E_{p1}=m_{1}gsinq_{1} \end{array} \end{array}$$ (6)

$$\begin{array}{c} \begin{array}{c} E_{p2}=m_{2}g\left(l_{1}sinq_{1}+d_{2}sin\left(q_{1}+q_{2}\right)\right) \end{array} \end{array}$$ (7)

The total potential energy of the exoskeleton single leg is:

$$\begin{array}{c} \begin{array}{c} E_{p}=E_{p1}+E_{p2} \end{array} \end{array}$$ (8)

### Derivation of Lagrange’s equation

The Lagrangian function is defined as:

$$\begin{array}{c} \begin{array}{c} L=E_{p}+E_{k} \end{array} \end{array}$$ (9)

*E*_*k*_ and *E*_*p*_ are the total kinetic and total potential energy of the exoskeleton single leg, *E*_*k*_ are a function of hip and knee angle *q*_*i*_(*i* = 1, 2) and angular velocity $\dot{q_{i}}\left(i=1,2\right)$, *E*_*p*_ is a function of hip and knee angle, so:

$$\begin{array}{c} \begin{array}{c} \tau_{i}=\frac{d}{dt}\frac{\partial L}{\partial q_{i}}-\frac{\partial L}{\partial q_{i}} \end{array} \end{array}$$ (10)

The exoskeleton dynamics equation can be obtained by finding the algebraic expression of each parameter in the above equation and substituting it into the partial differential operation:

$$\begin{array}{c} \begin{array}{c} D\left(q\right)\ddot{q}+C\left(q,\dot{q}\right)\dot{q}+G\left(q\right)=\tau \end{array} \end{array}$$ (11)

$$\begin{array}{c} D\left(q\right)=(\begin{array}{cc} \frac{1}{4}\left(m_{1}+m_{2}\right)l_{1}^{2}+\frac{1}{4}m_{2}l_{2}^{2}+m_{2}l_{1}l_{2}cosq_{2} & \frac{1}{4}m_{2}l_{2}^{2}+\frac{1}{2}m_{2}l_{1}l_{2}cosq_{2}\\ \frac{1}{4}m_{2}l_{2}^{2}+\frac{1}{2}m_{2}l_{1}l_{2}cosq_{2} & \frac{1}{4}m_{2}l_{2}^{2} \end{array}) \end{array}$$

$$\begin{array}{c} C\left(q,\ddot{q}\right)=(\begin{array}{cc} -m_{2}l_{1}l_{2}\dot{q_{1}}sinq_{2} & -\frac{1}{2}m_{2}l_{1}l_{2}\dot{q_{2}}sinq_{2}\\ \frac{1}{2}l_{1}l_{2}\dot{q_{1}}sinq_{2} & 0 \end{array}) \end{array}$$

$$\begin{array}{c} G\left(q\right)=(\begin{array}{c} \left(\frac{1}{2}m_{1}+m_{2}\right)gl_{1}cosq_{1}+\frac{1}{2}m_{2}gl_{2}cos\left(q_{1}+q_{2}\right)\\ \frac{1}{2}m_{2}gl_{2}cos\left(q_{1}+q_{2}\right) \end{array}) \end{array}$$

### RBF neural network optimization control

RBF neural network is a three-layer neural network with nonlinear mapping relationship from input to output, linear mapping relationship from hidden layer to output, and a neural network that locally approximates the target value. Compared with BF neural networks, RBF neural networks have the advantages of fast convergence speed and are not easy to fall into local minimum values [16]. For the real-time control of exoskeletons of nonlinear systems, RBF neural network is adopted to optimize the control parameters [17] to effectively improve the control accuracy, anti-interference and adaptability of the system.

**Fig 3:**
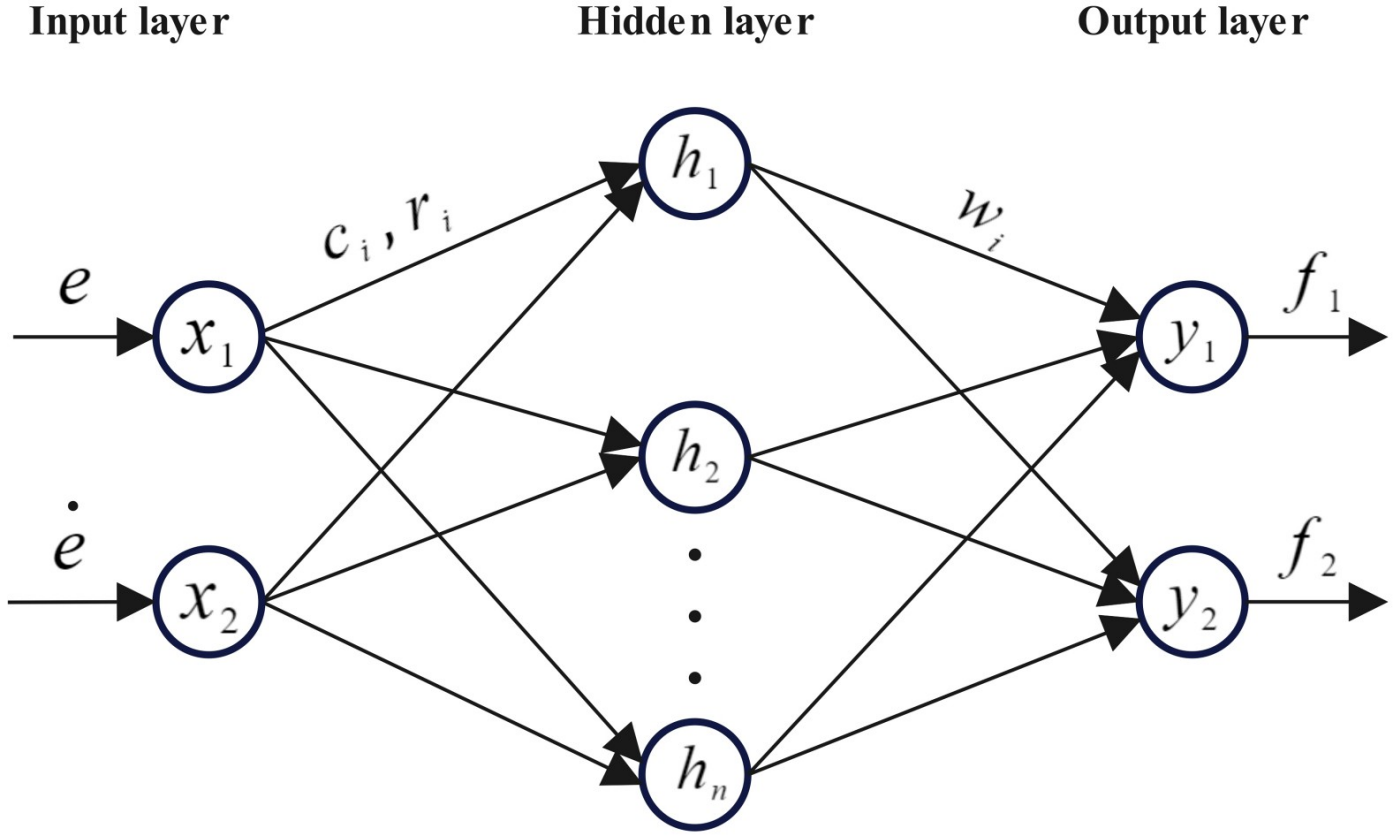
RBF neural network diagram.

As shown in Fig 3, the RBF neural network in this paper uses the mode of 2*13*2, and the input layer contains two input values, namely the exoskeleton hip and knee angle tracking error *e*_*i*_ (*i* = 1, 2) and the angular velocity tracking error $\dot{e_{i}}\left(i=1,2\right)$. The hidden layer contains a total of 13 neurons, and the activation function in the hidden layer uses a Gaussian function [18] which converts the input vector into a linearly separable form. The output layer contains two output values *f*_1_, *f*_2_, which represent the model control law parameters, and continuously adjust the neural network parameters to change the output value by feeding back the error values and, approaching the error minimum.

Suppose the input value be, which can be expressed as:

$$\begin{array}{c} \begin{array}{c} x={\left(e,\dot{e}\right)}^{T} \end{array} \end{array}$$ (12)

where *x* is the matrix of the angular tracking error *e*_*i*_ (*i* = 1, 2) and angular velocity tracking error $\dot{e_{i}}\left(i=1,2\right)$ of the exoskeleton hip and knee.

Using the Gaussian function, the input matrix *x* is mapped to a high dimension, which can be expressed as:

$$\begin{array}{c} \begin{array}{c} h\left(x\right)=exp\left(-\frac{||x-c_{i}\left|\right|}{r_{i}^{2}}\right) \end{array} \end{array}$$ (13)

where *c*_*i*_ and *r*_*i*_ represent the center and width of the Gaussky function respectively.

The neural network output can be expressed as:

$$\begin{array}{c} \begin{array}{c} y_{i}=h_{i}*w_{i} \end{array} \end{array}$$ (14)

where *y*_*i*_ represents the output of the neural network after the exoskeleton uncertainty term *f* is input into the controller.

### PSO finds the optimal weight

**Fig 4:**
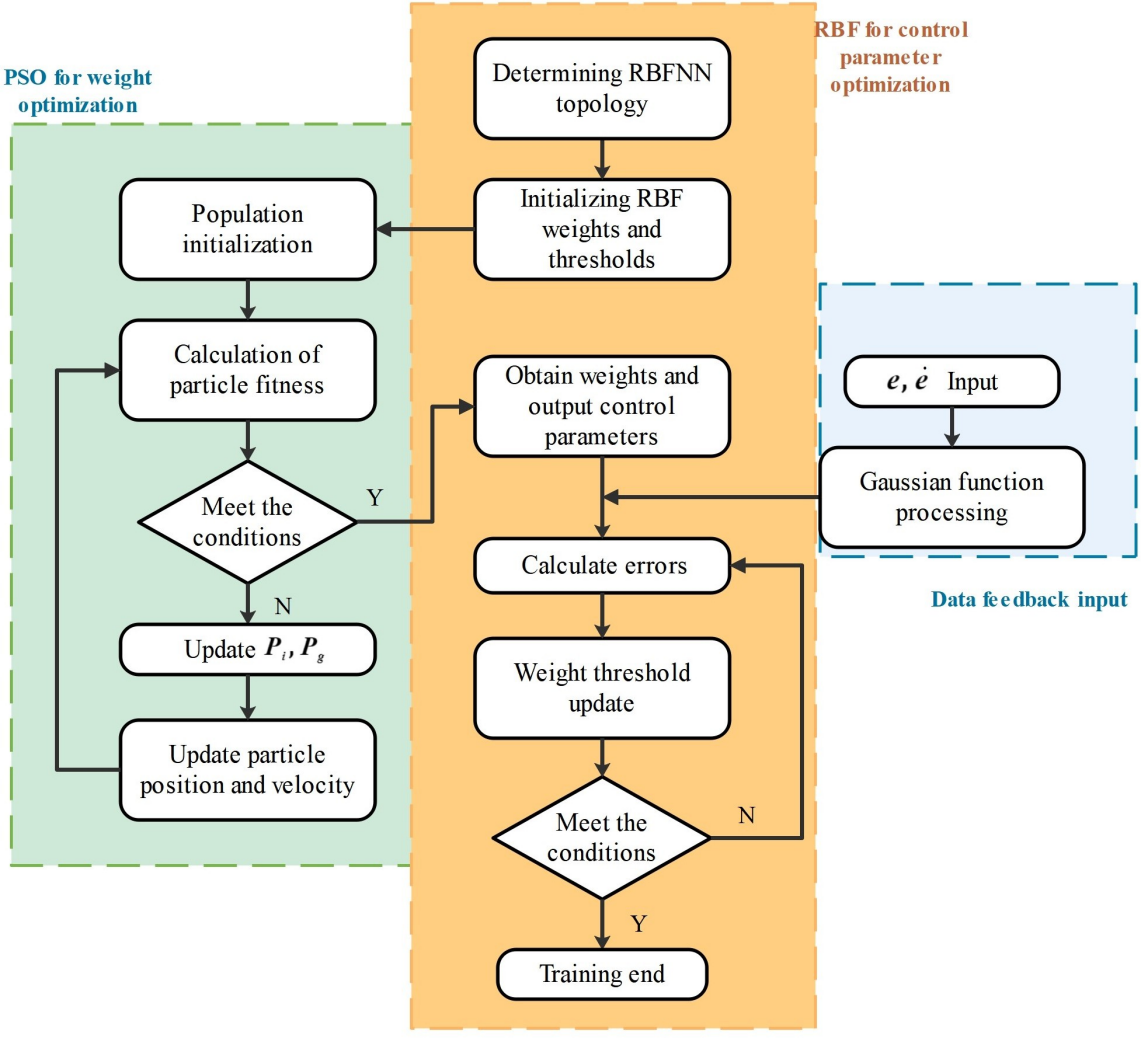
PSO optimization RBFNN weight flowchart.

PSO algorithm is a probabilistic global optimization search algorithm, and its core idea is to seek the optimal solution through cooperation and information sharing between individuals in the population [19]. In Section Derivation of kinetic equations, the RBF neural network optimization controller parameters are introduced, this section mainly introduces the PSO to seek the optimal weight of the neural network, and its optimization flow chart is as follows:

As shown in Fig 4, PSO seeks the optimal weight of the neural network, first determines the neural network topology, and then sets the RBF weight range. BSF weights have a total of 26 dimensions, with a lower weight limit of 10 and an upper limit of 50. The value of the weight can be regarded as the population initialization standard, the limit domain of the particle position is set to the upper and lower limit values of the weight [10, 50], and the particle velocity is limited to between -2 and +2. The minimum objective function is constructed by system input error $e_{i},\dot{e_{i}}\left(i=1,2\right)$, and the fitness function is further constructed, so as to complete the calculation of the particle swarm fitness function [20].

The minimum objective function is as follows:

$$\begin{array}{c} \begin{array}{c} J=\sum_{i=1}^{n}|e_{i}|+\sum_{i=1}^{n}\left|\dot{e_{i}}\right| \end{array} \end{array}$$ (15)

Converted into an fitness function:

$$\begin{array}{c} \begin{array}{c} F=\frac{1}{J} \end{array} \end{array}$$ (16)

The particle fitness function and the limit range of particle swarm velocity and position are used to continuously iterate the particle swarm, continuously update the particle position and velocity, seek the minimum error value, output the optimal neural network weight, and accelerate the convergence speed of the neural network.

### PSO-RBFNN combined to optimize torque control

**Fig 5:**
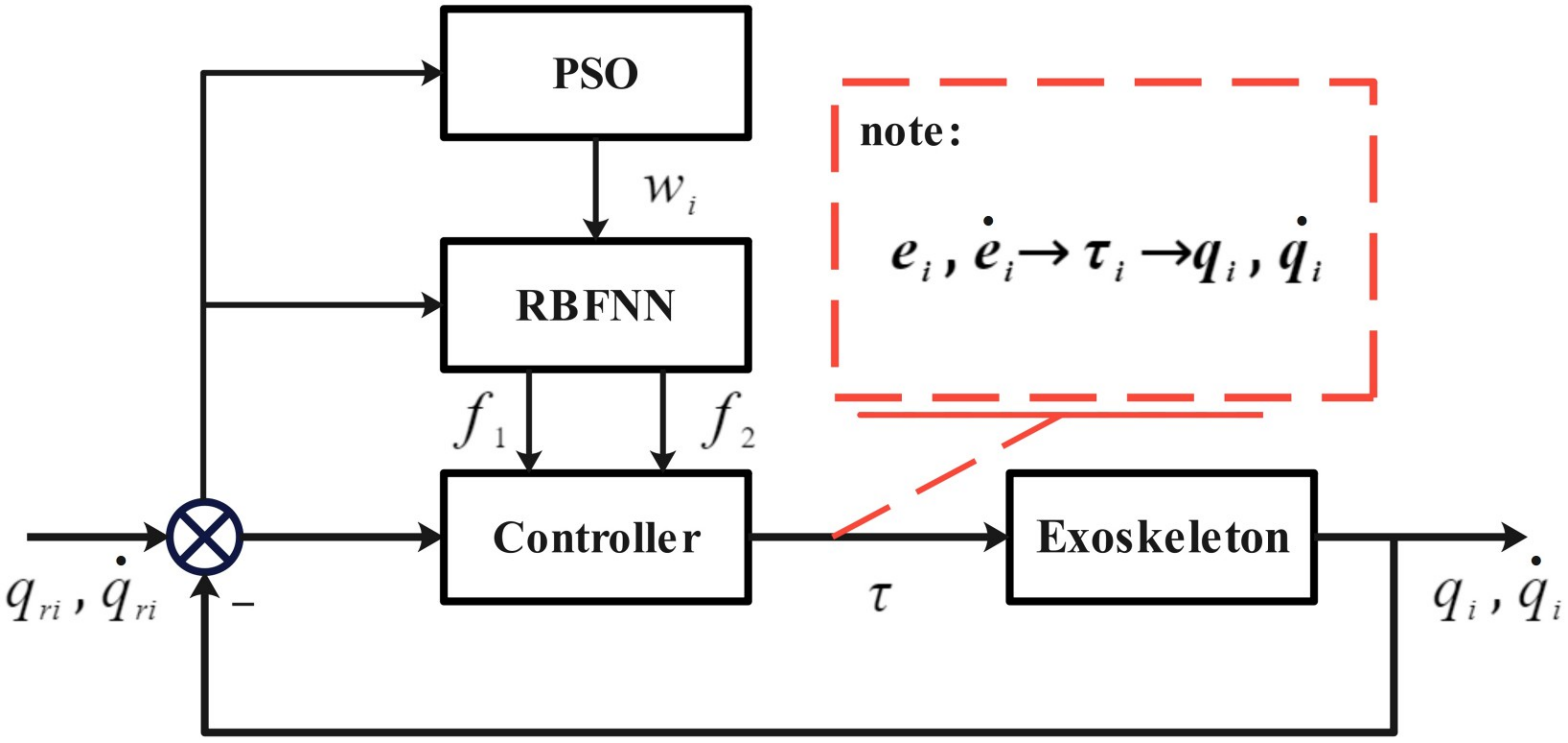
PSO-RBFNN optimal control.

Sections RBF neural network optimization control and PSO finds the optimal weight introduce the PSO and RBFNN optimization process, in which the RBF neural network continuously adjusts the output control parameters to further approximate the minimum error value, and the PSO algorithm obtains the weight of the neural network and enters it into RBF to achieve the purpose of accelerating the approximation speed. The exoskeleton control of PSO-RBFNN combined optimization can approach the minimum error value deeper and faster, realize accurate real-time control of exoskeleton, and the overall control process is shown in the figure below:

As shown in Fig 5, $q_{i},\dot{q_{i}}\left(i=1,2\right)$ represents the output angle and angular velocity of the exoskeleton hip and knee joints. Through closed-loop negative feedback control [21], the system input value is subtracted from the output value to obtain error $e_{i},\dot{e_{i}}\left(i=1,2\right)$, error $e_{i},\dot{e_{i}}$ is used as the minimum target value of PSO-RBFNN optimization, PSO-RBFNN continuously adjusts the network weight, outputs the value of the appropriate model uncertain *f* term, substitutes it into the controller, and the Controller design in the figure is as follows.

According to the derived exoskeleton dynamics Eq 11, if the model is accurately modeled, the control law of the controller can be designed as:

$$\begin{array}{c} \begin{array}{c} \tau =D\left(q\right)\left(\ddot{q_{r}}-k_{1}\dot{e}-k_{2}e\right)+C\left(q,\dot{q}\right)\dot{q}+G\left(q\right) \end{array} \end{array}$$ (17)

In the actual motion, it is difficult to obtain the accurate model of the controller, and the inaccuracy of the model will lead to the degradation of control performance [22], so it is necessary to identify the inaccurate part of the model, suppose the model uncertainty term be:

$$\begin{array}{c} \begin{array}{c} f=D_{0}^{-1}\left(\Delta D\ddot{q}+\Delta C\dot{q}+\Delta G\right) \end{array} \end{array}$$ (18)

The neural network outputs the actual *f* value, which has an important impact on the torque control performance of the exoskeleton. Substitute *f* into the controller to get the corrected control law:

$$\begin{array}{c} \begin{array}{c} \tau =D\left(q\right)\left(\ddot{q_{r}}-k_{1}\dot{e}-k_{2}e\right)+C\left(q,\dot{q}\right)\dot{q}+G\left(q\right)-D\left(q\right)f \end{array} \end{array}$$ (19)

Therefore, the relationship between the actual state of the exoskeleton and the control force can be obtained, and the error of the angle and angular velocity value and the expected value returned by the sensor can be input into the controller, and the motor continuously adjusts the output torque to make the exoskeleton trajectory close to the ideal input curve.

## Results

### Qualitative and quantitative analysis of results

**Fig 6:**
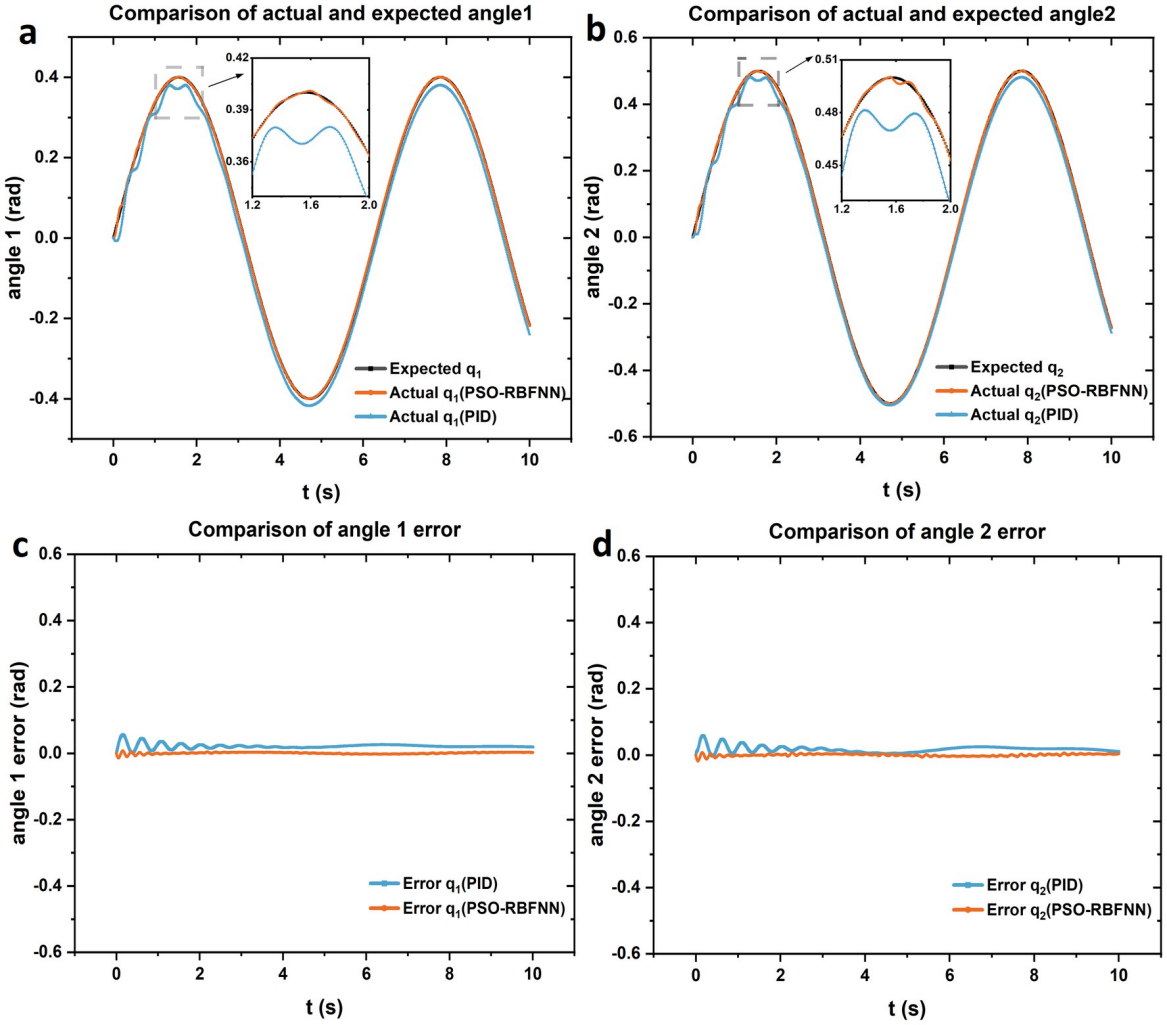
Joint trajectory and error.

According to the above derivation, the MATLAB design m-function is used to carry out simulation experiments, and the expected trajectories and expected angular velocities of the hip angle *q*_1_ of the exoskeleton are set to $q_{1}=0.4sint,\dot{q_{1}}=0.4cost$, and the expected trajectories and expected angular velocities of knee angle *q*_2_ are $q_{2}=0.5sint,\dot{q_{2}}=0.5cost$ respectively. The expected trajectory and expected angular velocity were taken as the input of the exoskeleton system, and the joint angular torque control was optimized by PSO-RBFNN, and the relationship between the input value and the output value of the system was obtained. As shown in Fig 6, taking the joint trajectory as an example, the relationship between the input angle and the output angle of the exoskeleton and the error value is obtained through simulation, and the PSO-RBFNN is compared with another superior control method, PID control, and the comparison results show that the optimized torque control of PSO-RBFNN has a better control effect.

Fig 6 shows the trajectory tracking curve and angle error curve of joint angles *q*_1_ and *q*_2_, as shown in the figure, the torque control optimized by PSO-RBFNN only has a slight fluctuation in the output trajectory before 1.5 s, and then the output trajectory can track the desired trajectory well, and the error value is almost 0. In contrast, PID control, the initial fluctuation is great, after stabilization, the effect of output trajectory tracking the desired trajectory is general, compared with the PSO-RBFNN optimization control, the error of PID control is also larger.

Comparing the control performance standards of the two control methods, the optimized torque control of PSO-RBFNN has better stability and speed than PID control. As can be seen from Fig 6a and 6c, by entering the desired trajectory at the same time, the PSO-RBFNN fluctuations are smaller and can stabilize more quickly. It can be seen from Fig 6b and 6d that the error of PSO-RBFNN is almost 0 after 1.5 s, but the error of PID control is large, obviously, PSO-RBFNN has better accuracy. Therefore, compared with PID control, the exoskeleton torque control optimized by PSO-RBFNN has advantages in control performance.

**Fig 7:**
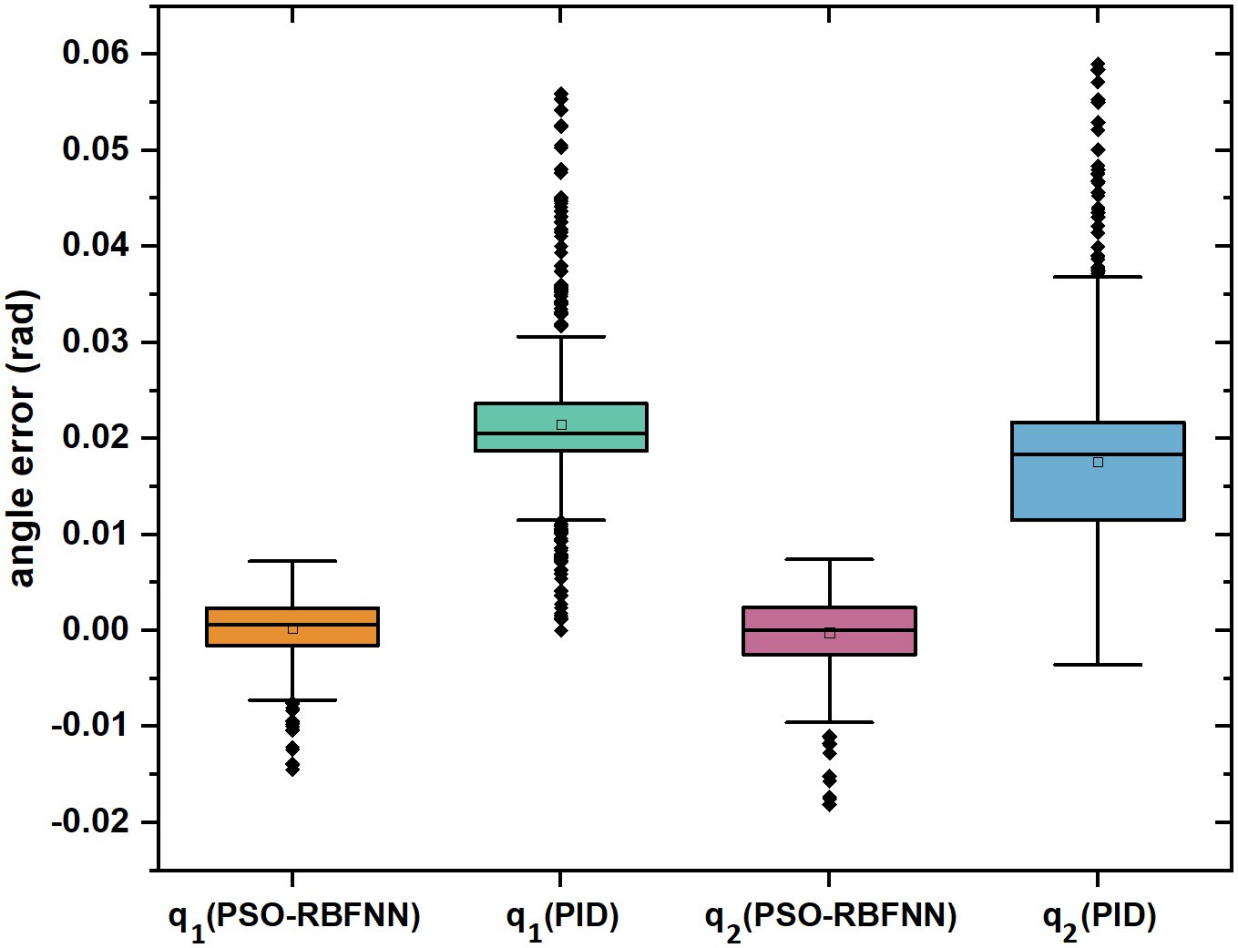
Quantitative analysis chart of error.

To make the analysis more specific and reliable, the control results are quantitatively analyzed after qualitative analysis, as shown in Fig 7 and Table 2:

**Table 2:** Comparison of error benchmarks.

Object	Average	Max	Stable time
*q*_1_ (PSO-RBF)	5.2*10–4 rad	0.007 rad	About 1 s
*q*_1_ (PID)	0.021 rad	0.03 rad	About 3 s
*q*_2_ (PSO-RBF)	5.9*10–5 rad	0.009 rad	About 1 s
*q*_2_ (PID)	0.018 rad	0.036 rad	About 4 s

Table notes the comparison of error benchmarks.

The error block diagram and error values are shown in Fig 7 and Table 2. This paper compares the average error, error range and settling time under the two different control methods using PID control as the benchmark., and further explore the superiority of torque control based on PSO-RBFNN optimization based on these specific data. In terms of stability, using the PSO-RBFNN optimized torque control, the error intervals of *q*_1_ and *q*_2_ are [0, 0.007] and [0, 0.009], which is less volatile than [0, 0.03] and [0, 0.036] under pid control, and quickly tends to 0 degrees and no longer fluctuates. In terms of accuracy, using the torque control optimized by PSO-RBFNN, the average angular errors of *q*_1_ and *q*_2_ are 5.2*10–4 rad and 5.9*10–5 rad. The average angular errors under PID control are 0.021 rad and 0.018 rad, which are much larger than the errors under PSO-RBFNN optimization. By comparing the stability time of PSO-RBFNN optimization and PID control to compare the rapidity of the two, after PSO-RBFNN optimization, the angle output trajectory of exoskeleton joints is basically stable after about 1 s, with only slight fluctuations however, after PID control, the joint output trajectory remained unstable after 3 s, accompanied by a certain range of fluctuations. In summary, the experimental results take the trajectory error of the exoskeleton under PID control as the benchmark, and verify that the rehabilitated exoskeleton torque control optimized by PSO-RBFNN has good performance in stability, accuracy and rapidity.

### Analysis of the influence of uncertainties

In the practical application of exoskeleton, due to the interference of various external factors, the exoskeleton control will be uncertain, which in turn affects the training and rehabilitation effect of patients. Therefore, the analysis of uncertainties in exoskeleton control is particularly important to verify the robustness of exoskeleton control optimized by PSO-RBFNN in practical applications. Firstly, this paper needs to determine the main interference factors in the actual rehabilitation of exoskeletons, which is the interaction of the human body when wearing exoskeleton, which will cause the exoskeleton to be interfered with additional load, and then change the internal parameters of the exoskeleton model and affect the control effect.

In order to analyze the control effect of exoskeleton under the influence of interference factors, this paper simulates the exoskeleton control trajectory under human wear, and compares it with that without interference to verify its robustness. The simulation operation is: keep the input trajectory unchanged, add the thigh mass and calf mass of the exoskeleton to the human load increment, adjust the total thigh weight and calf total weight of 20 kg and 15 kg, and study the change of tracking trajectory after interference.

**Fig 8:**
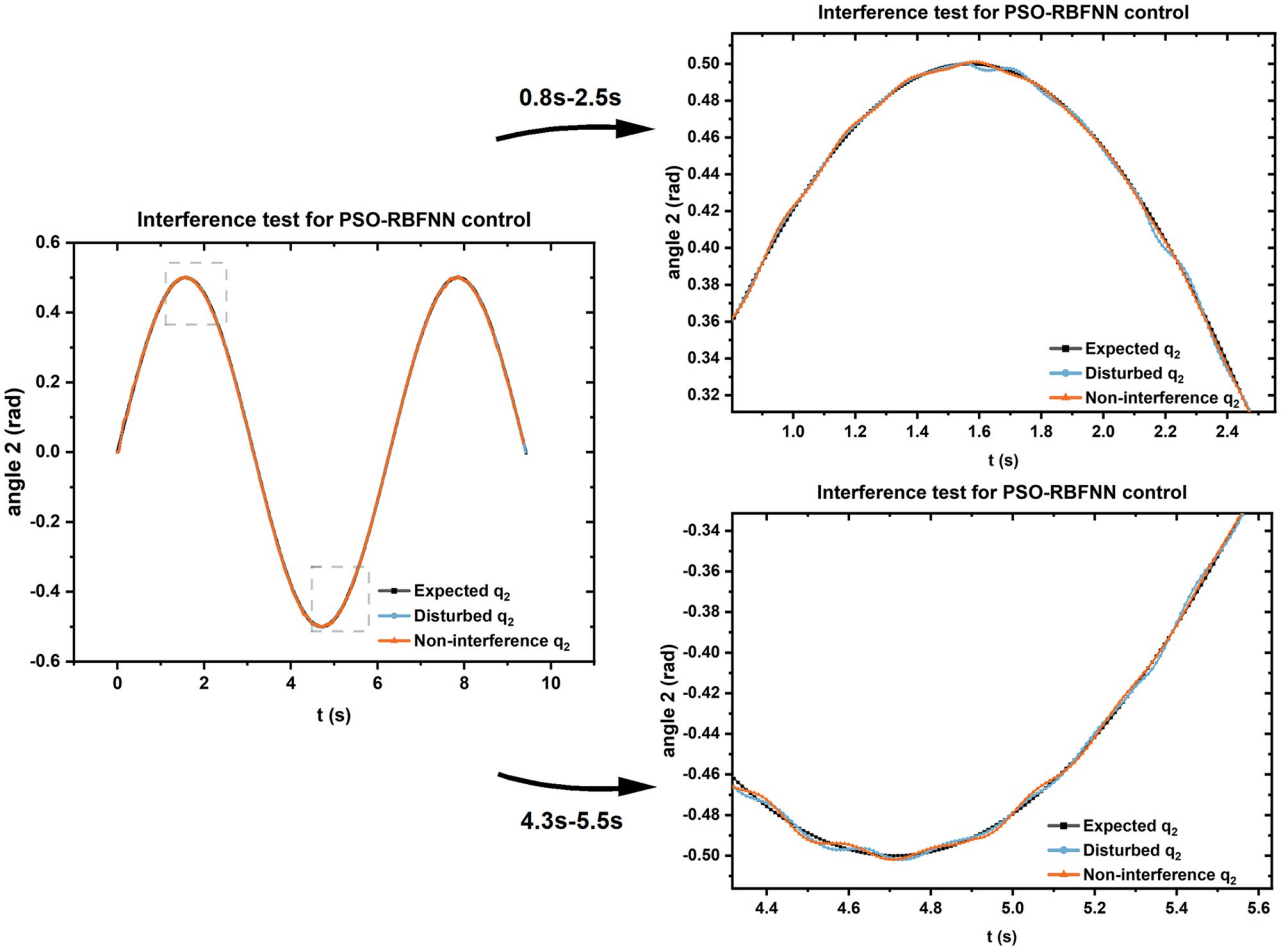
Interference test chart.

As shown in Fig 8, this paper takes joint angle 2 as an example to show the trajectory curve of the exoskeleton after being subjected to human interaction, and compares it with the undisturbed trajectory. It can be found that the trajectory error fluctuation is not large after adding the influence of human load, and it is still consistent with the expected trajectory, which can verify the anti-interference of the exoskeleton control optimized by PSO-RBFNN to the outside world under the actual action.

## Conclusion

Exoskeletons are widely used in the field of medical rehabilitation, and in this paper, the control of exoskeletons is studied in view of the rehabilitation training effect of exoskeletons. Due to the wide application of deep learning algorithms in the field of optimization, this paper designs a control method for optimizing exoskeleton torque control jointly by PSO and RBFNN, and after comparing with PID control, it is found that the performance of PSO-RBFNN optimized torque controller is superior to that of PID controller, the actual motion trajectory tracking effect of joint angle is better, the control speed is faster, the stability is higher and the results are more accurate. Therefore, it is concluded that the exoskeleton torque control optimized by PSO-RBFNN has a better effect and more advantages in patient rehabilitation training. Moreover, the method proposed in this paper is universal, and the optimized exoskeleton can not only be applied to the field of rehabilitation, but also has good results in manufacturing and logistics and transportation.

## Supporting information

S1 File (RAR)

S2 File (XLSX)
